# Prognostic Relevance of Objective Response According to EASL Criteria and mRECIST Criteria in Hepatocellular Carcinoma Patients Treated with Loco-Regional Therapies: A Literature-Based Meta-Analysis

**DOI:** 10.1371/journal.pone.0133488

**Published:** 2015-07-31

**Authors:** Bruno Vincenzi, Massimo Di Maio, Marianna Silletta, Loretta D’Onofrio, Chiara Spoto, Maria Carmela Piccirillo, Gennaro Daniele, Francesca Comito, Eliana Maci, Giuseppe Bronte, Antonio Russo, Daniele Santini, Francesco Perrone, Giuseppe Tonini

**Affiliations:** 1 Medical Oncology, University Campus Bio-Medico, Rome, Italy; 2 Clinical Trials Unit, National Cancer Institute, Naples, Italy; 3 Department of Surgical, Oncological and Oral Sciences, University of Palermo, Palermo, Italy; 4 Department of Oncology, University of Turin, San Luigi Gonzaga Hospital, Regione Gonzole 10, 10043 Orbassano (TO), Italy; Yonsei University College of Medicine, KOREA, REPUBLIC OF

## Abstract

**Background:**

The European Association for the Study of the Liver (EASL) criteria and the modified Response Evaluation Criteria in Solid Tumors (mRECIST) are currently adopted to evaluate radiological response in patients affected by HCC and treated with loco-regional procedures. Several studies explored the validity of these measurements in predicting survival but definitive data are still lacking.

**Aim:**

To conduct a systematic review of studies exploring mRECIST and EASL criteria usefulness in predictive radiological response in HCC undergoing loco-regional therapies and their validity in predicting survival.

**Methods:**

A comprehensive search of the literature was performed in electronic databases EMBASE, MEDLINE, COCHRANE LIBRARY, ASCO conferences and EASL conferences up to June 10, 2014. Our overall search strategy included terms for HCC, mRECIST, and EASL. Loco-regional procedures included transarterial embolization (TAE), transarterial chemoembolization (TACE) and cryoablation. Inter-method agreement between EASL and mRECIST was assessed using the k coefficient. For each criteria, overall survival was described in responders vs. non-responders patients, considering all target lesions response.

**Results:**

Among 18 initially found publications, 7 reports including 1357 patients were considered eligible. All studies were published as full-text articles. Proportion of responders according to mRECIST and EASL criteria was 62.4% and 61.3%, respectively. In the pooled population, 1286 agreements were observed between the two methods (kappa statistics 0.928, 95% confidence interval 0.912–0.944). HR for overall survival (responders versus non responders) according to mRECIST and EASL was 0.39 (95% confidence interval 0.26–0.61, p<0.0001) and 0.38 (95% confidence interval 0.24–0.61, p<0.0001), respectively.

**Conclusion:**

In this literature-based meta-analysis, mRECIST and EASL criteria showed very good concordance in HCC patients undergoing loco-regional treatments. Objective response according to both criteria confirms a strong prognostic value in terms of overall survival. This prognostic value appears to be very similar between the two criteria.

## Introduction

Hepatocellular carcinoma (HCC) represents today the fifth most common cancer diagnosis and the third most common cause of cancer-related deaths [1]. Several risk factors have been identified, including chronic hepatitis B and/or C viral infections, some inherited errors of metabolism (i.e. hemocromatosis, Wilson’s disease, α_1_-antitrypsin deficiency), primary hepatic immune disease and primary biliary cirrhosis [2]. More recently, a higher risk of liver cancer development has also been reported in patients affected by systemic metabolic syndrome, diabetes mellitus and non-alcoholic fatty liver disease [3]. Since 60%-80% of patients with newly diagnosed HCC have cirrhosis of the liver, ultrasonography and AFP testing every 6–12 months are routinely performed to promote an early detection of malignant nodule transformation in asymptomatic patients. Despite screening programs fewer than 20% of HCC are curable at the time of diagnosis and, given the presence of co-existent chronic liver disease in most cases, valuation of the underlying liver function is essential in therapeutical decision, since it can affect treatment efficacy and influence tolerability profile [4]. Current guidelines from the American Association for the Study of Liver Diseases for intermediate-stage HCC recommend loco-regional approaches for those patients with localized disease not suitable for surgical resection/transplantation [5]. By inducing alteration in local temperature (radiofrequency ablation, microwave ablation, cryoablation) or determining selective catheter-based infusion of particles in cancer supplying arterial branches (chemoembolization), these procedures lead to tumor necrosis and ensure disease control [6]. Radiological response is a well-recognised surrogate endpoint in the assessment of treatment efficacy in phase II studies, whereas survival remains crucial for phase III [7]. However conventional response evaluation criteria (WHO, World Health Organization and RECIST, Response Evaluation Criteria in Solid Tumors) have shown poor correlation with survival outcome in HCC patients, since they do not address measures of antitumor activity other than tumour shrinkage (which is based on the sum of bidimensional measurements of target lesions) [8]. To overcome this limitation, a modification of the response assessment was developed starting from 2001 in order to include the concept of tumour viability (tumoral tissue showing arterial uptake in the arterial phase of contrast-enhanced imaging techniques) and discriminate treatment efficacy from early failure [6]. The European Association for the Study of the Liver (EASL) criteria and the modified Response Evaluation Criteria in Solid Tumors (mRECIST) were adopted in the evaluation of radiological response in patients affected by HCC and treated with loco-regional procedures. EASL and mRECIST criteria differ from each others in terms of number of target lesions (all versus < = 2) and calculation method (bidimensional versus unidimensional) as reported in Table 1. Several studies [9, 10]indicate that evaluating the largest two lesions is generally the most useful procedure for measuring TACE responses under both EASL and mRECIST, even if the optimal number of lesions is not formally indicated in mRECIST criteria.

**Table 1:** Comparison between mRECIST and EASL criteria for HCC^10^.

mRECIST criteria	EASL criteria
• **CR:** No intratumoral arterial enhancement in all target lesions.	• **CR:** Absence of any viable lesions (enhancing lesion on arterial phase of T1 post-contrast sequence on dynamic abdominal magnetic resonance imaging study).
• **PR:** ≥ 30% reduction of the sum of diameters of viable (enhancement in the arterial phase) target lesions.	• **PR:** ≥ 50% reduction of the sum of diameters of viable target lesions.
• **SD:** Features classifiable as neither partial response nor progressive disease.	• **SD:** Features classifiable as neither partial response nor progressive disease.
• **PD:** ≥ 20% increase of the sum of the diameters of viable target lesions.	• **PD:** ≥ 25% increase of the sum of the diameters of viable target lesions.

**Abbrevation:** HCC, hepatocellular carcinoma; mRECIST, modified Response Evaluation Criteria in Solid Tumors; CR, complete response; PR, partial response; SD, stable disease; PD, progressive disease.

Up to now no large prospective validation is available for both mRECIST and EASL criteria and further studies are needed to confirm the validity of these measurements and their correlation with survival. Here we present a literature-based review gathering together all published retrospective studies comparing mRECIST and EASL criteria predictivity of tumor response and survival outcomes.

## Methods

### Selection of studies

Study selection was conducted according to the preferred reporting items for systematic reviews and meta-analyses (PRISMA) statement [11] (S1 PRISMA Checklist). A comprehensive search of the literature was performed in electronic databases EMBASE, MEDLINE and COCHRANE LIBRARY, from February 2010 up to June 2014. The references within the identified articles were then manually searched for additional studies. Our overall search strategy included terms for HCC, mRECIST, and EASL. To be eligible for inclusion, studies had to met the following criteria: (1) Loco-regional procedures included transarterial embolization (TAE), transarterial chemoembolization (TACE) and cryoablation, (2) response assessment after loco-regional treatments was evaluated according to both mRECIST and EASL criteria, (3) availability of data about overall survival and (4) reported k coefficient as measurement of mRECIST and EASL concordance, or availability (in the text or in a table) of the information needed to calculate it. Moreover, meeting abstracts presented in the most recent International Meetings (American Society of Clinical Oncology, American Association for the Study of Liver Diseases, and European Association for the Study of the Liver), personal presentation and no published data from ongoing study were explored and included if the above criteria were respected. No language limitation were observed, as all selected works were written in English.

### Data extraction, clinical end points and quality assessment

By reading the full texts of the selected citations, two investigators (M.S. and L.D.) independently evaluated each identified for eligibility and quality, and then extracted the following data: name of all authors, year of publication, number of enrolled patients, type of loco-regional treatment, reported hazard ratio (HR) for overall survival (OS) according to mRECIST and EASL criteria and k coefficient of concordance in each study. Since progression free survival was not available for all studies, differently from overall survival outcome, these data were not extrapolated.

### Statistical analysis

After data were abstracted, the authors proceed to their analysis using Review Manager (RevMan 5), the software used for preparing and maintaining Cochrane Reviews. Inter-methods concordance between similar categorical items of the two criteria was measured using the k coefficient. The strength of agreement based on k values was interpreted as follows: k less than 0.21, poor; k of 0.21–0.40, fair; k of 0.41–0.60, moderate; k of 0.61–0.80, good; and k greater than 0.80, excellent [12]. Hazard ratio (HR) for overall survival were used for meta-analysis, considering responders (complete or partial response) versus non responders patients, using the generic inverse variance outcome type in RevMan. To account for the heterogeneity of studies, a random-effects model was applied. For both mRECIST and EASL criteria, funnel plots were used to grossly exclude publication bias.

## Results

**Fig 1:**
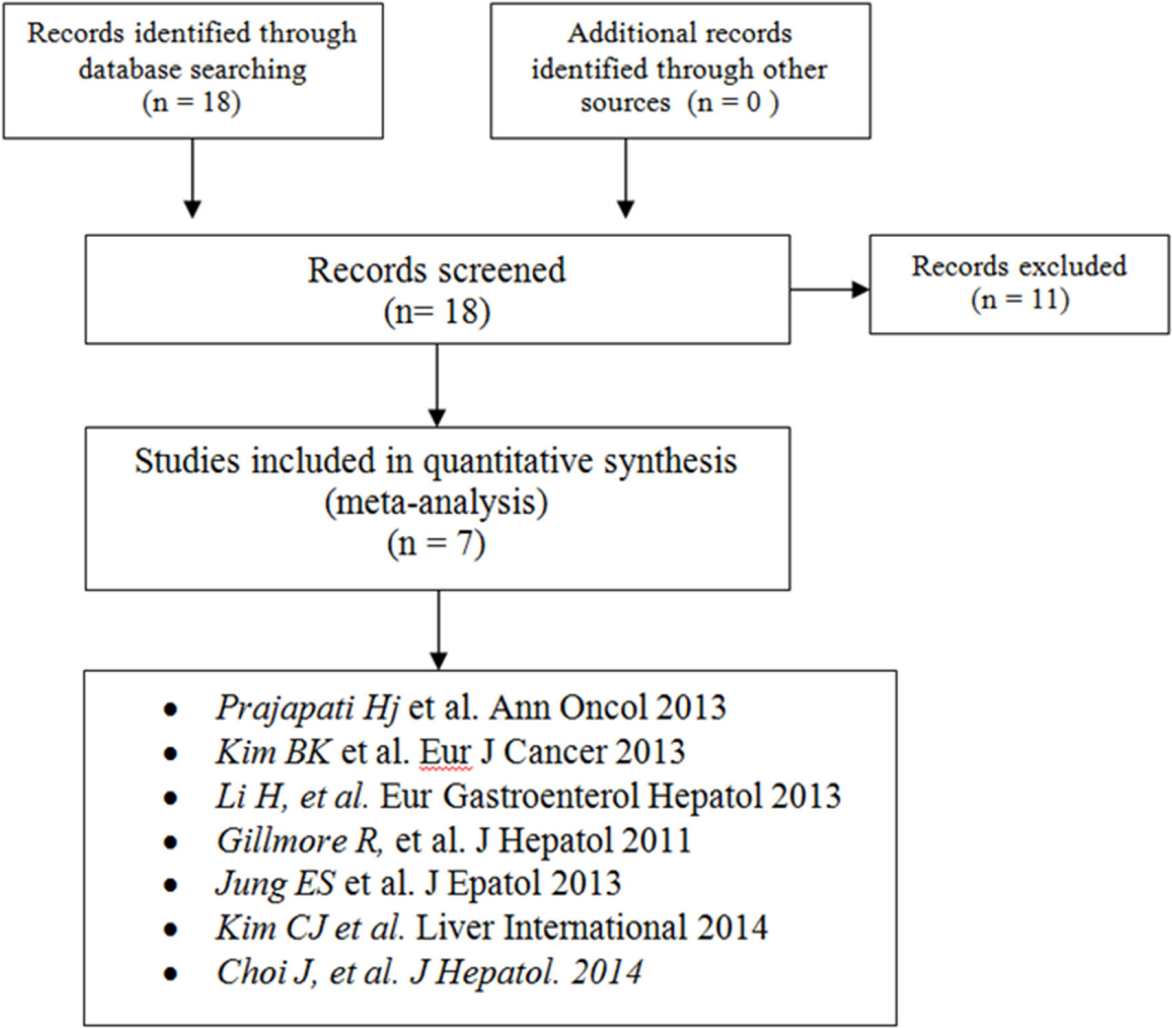
Flow diagram of study selection.

Of 18 titles identified in the original search, 7 reports including 1357 patients were considered eligible for analysis (Fig 1). All trials, available as full-text articles, have been conducted retrospectively. The overall collection data period was March 2000 –June 2014. Diagnosis of HCC was confirmed by biopsy or radiologic imaging techniques according to the guidelines in each study.

11 studies were considered not eligible for analysis, as reported in Table 2. One was focused on the prognostic role of number of target lesions more than on that of response criteria [13]. Another study reported response and survival data at specific time point, so that it was not possible to extract the overall data [14]. In five works authors chose RECIST 1.0, RECIST 1.1 or volumetric RECIST version as comparator response criteria [15–19]. Lee IJ et al focused on correlation between radiologic response (according to WHO, RECIST, mRECIST and EASL criteria) and pathologic post resection viability [20]. Finally three studies compared the prognostic value of all known criteria (WHO, RECIST, mRECIST and EASL) to each other but it was not possible to draw all parameter we need for analysis [10, 21, 22]. However, data from one of these excluded studies [19] were obtained from a second, more recent publication [23] describing the same series of patients.

**Table 2:** Study excluded from metanalysis.

*Kim BK*, *et al*. *Clin Cancer Res*. *2013*
*Boatta E*, *et al Indian J Radiol Imaging*. *2013*
*Shuster A*, *et al J Vasc Interv Radiol*. *2013*
*Price TR*, *et al Cancer*. *2012*
*Riaz A*, *et al JAMA*. *2010*
*Lin M*, *et al J Vasc Interv Radiol*. *2012*
*Yozo Saro*, *et al Upsala Journal of Medical Sciences*. *2013*
*Lee IJ*, *et al Gastrointestinal Cancers Symposium J Clin Oncol 2013*
*Shim JH*, *et al Radiology*. *2012*
*Meza-Junco J*, *et al Cancer Treat Rev*. *2012*
*Duke E*, *et al J Vasc Interv Radiol*. *2010*

Baseline patients characteristics were homogeneous among retrospective cohorts and are listed in Table 3. General exclusions criteria were: inadequate target lesion (infiltrative pattern or largest lesion <1 cm); (2) presence of an additional primary malignancy in other organ; (3) presence of extrahepatic lesions; (4) presence of uncontrolled functional or metabolic disease. Of note, only two studies [13, 22] limited the inclusion to Child-Pugh class A patients, while the other studies included a minority of Child-Pugh B patients, and even a small percentage of Child-Pugh C patients in 1 case [9]. Most enrolled patients underwent TACE as initial therapy and then repeated TACE on demand at 4–8 weeks after the first cycle. One study [9] used drug-eluting beads (DEB) TACE. In all cases femoral artery approach was preferred. Arteries selective catheterization was followed by TACE following the internal guidelines of the different institution. Chemotherapeutics agents were doxorubicin (in three studies [9, 23, 24] or cisplatin (in two studies [22, 25]. Only one among the considered studies used cryoablation as loco-regional procedure [26]. Tumour measurements were performed according to the EASL and mRECIST criteria and assessment of response was carried out by contrast-enhanced spiral computed tomography (CT) or gadolinium-enhanced magnetic resonance imaging (MRI) after 4–8 weeks from treatment, depending on each study. In detail, as reported in Table 4, the procedure used was CT in the majority of cases, with the exception of one study using MRI [9] and two studies [13, 25] using CT or MRI (mostly CT in the study by Gillmore *et al*; no further details in the study by Kim BK *et al*). As shown in Table 4, most studies considered the overall response, while the study by Kim CJ et al [27] reported only the index lesion, that is currently validated for follow-up of patients with HCC [15] (and it is recommended by the American Association for the Study of Liver Diseases).

**Table 3:** Baseline patient characteristics.

	Age	Sex	ECOG PS	Child-Pugh	BCLC stage	Tumor number	Treatment
**Gillmore**	67	M: 72	0: 43	A: 69	A: 6	1: 30	TAE: 57
		F: 11	1: 25	B: 13	B: 38	≥ 2: 53	TACE: 26
			2: 11	nr: 1	C: 36		
**Jung ES**	59.6	M: 85	0: 24	A: 77	A: 37	1: 46	TACE: 114
		F: 16	1: 66	B: 21	B: 38	≥ 2: 40	
			2: 8	nr: 1	C: 23		
**Kim BK**	60	M: 290	0: 190	All Child A	Not reported	1: 39	TACE: 292
		F: 42	1: 102			≥ 2: 253	
**Prajapati HJ**	61.7	M: 95	0: 49	A: 71	A: 14	1: 58	DEB TACE: 120
		F: 25	1: 56	B: 41	B: 20	≥ 2: 62	
			2: 15	C: 8	C: 76		
					End: 10		
**Li H**	60.5	M: 51	0: 44	A: 40	A: 4	Not reported	Cryoablation: 64
		F: 13	1: 8	B: 24	B: 30		
			2: 12		C: 30		
**Kim CJ**	62.9	M: 283	0: 332	A: 249	A: 152	1: 176	TACE: 368
		F: 85	1: 29	B: 118	B: 82	≥ 2: 192	
			2: 7	C: 0	C: 134		
**Choi J**	62	M: 290	Not reported	All Child A	All BCLC B	2–3: 197	TACE: 332
		F: 42				≥ 4: 135	

**Abbreviation:** ECOG PS: Eastern cooperative oncology group performance status, BCLC: Barcelona Clinic Liver Cancer

**Table 4:** Timing of instrumental assessment and lesions considered.

Reference	Exam	Timing	Response considered
Gillmore R, et al	84% CT, 16% MRI	Median 64 days (range 18–129)	Overall response
Li H, et al	CT	Median 40 days (range, 26–80)	Overall response
Prajapati HJ, et al	MRI	The median period between the DEB TACE therapies and post-treatment assessment MRI scans was 33.5 days (range 0–113). In 61.7% (n = 74) of patients, MRI scans (to assess treatment response) were carried out after first DEB TACE with a median period of 33.50 days. In 33.3% (n = 40) of patients, MRI scans were carried out after second DEB TACE with the median period of 35 days. In 5% (n = 6) of patients, post-treatment response was assessed using MRI scan carried out after third DEB TACE (median of 27 days).	Overall response
Kim BK, et al	CT or MRI	Treatment responses were assessed 4 weeks after the initial TACE.	Overall response
Jung ES, et al	CT was performed at baseline and 3–4 weeks after TACE, and wasused for response assessment. When indicated, 15 (15.3%) patientsunderwentprimovist-enhanced dynamic MRI to further clarifytumor viability.	We compared treatment responses between baselineimaging at diagnosis and follow-up imaging at early time point after 1–2 sessions of TACE.	Overall response
Kim CJ, et al	CT	Imaging follow-up (and hencemeasurement of the target lesions) was performed at 1 month following each treatment; subsequent scans were performed at scheduled 2 to 3 month intervals as per standard of care.For this analysis, even if several tumours were targeted during the first or subsequent treatments with chemoembolization or RFA, only the primary target lesions were used to assess response and followed longitudinally, even if those were not thelesions most recently treated.	Target response (overall not reported)
Choi J, et al	CT	1 month after the first TACE	Overall response

CT: computed tomography; MRI: magnetic resonance imaging; TACE: trans-arterial chemo-embolization; DEB TACE: drug-eluting beads trans-arterial chemo-embolization; RFA: radiofrequency ablation.

### Response

Both criteria embraced the following four response categories: complete response (CR), partial response (PR), stable disease (SD) and progressive disease (PD). Objective response (OR) included both CR and PR. As reported in Table 1, according to RECIST, CR was defined as the absence of arterially enhanced areas; PR and PD, as a greater than 30% decrease and a greater than 20% increase, respectively, of the sum of the longest diameters of the enhancing target lesions; and SD, as neither PR nor PD. According to EASL criteria, PR and PD were defined as a greater than 50% decrease and a greater than 25% increase, respectively, of the sum of the cross products of the enhancing target lesions. The appearance of new HCC lesions denoted PD under both criteria, confirmed when their diameter exceeded 1 cm or when the lesion became at least 1 cm larger on progressive scans.

Table 5 shows the response assessed with EASL criteria after loco-regional therapy administration in the 7 considered studies. Table 6 shows the response assessed with mRECIST criteria after loco-regional therapy administration in the same studies.

**Table 5:** Response assessment according to EASL criteria.

**References**	**N° Pts**	**CR**	**PR**	**SD**	**PD**
**Gillmore R, et al**	83	17 (20%)	32 (38%)	12 (14%)	22 (27%)
**Li H, et al**	64	10 (15,6%)	27 (42,2%)	18 (38,1%)	9 (14,1%)
**Prajapati HJ, et al**	120	24 (20%)	23 (19,2%)	40 (33,3%)	33 (27,5%)
**Kim BK, et al**	292	113 (38,7%)	106 (36,3%)	62 (21,2%)	11 (3,8%)
**Jung ES, et al**	114	34 (34,7%)	34 (34,7%)	25 (25,5%)	5 (5,1%)
**Kim CJ, et al**	368	162 (44,0%)	80 (21,7%)	59 (16.0%)	67 (18,2%)
**Choi J et al**	332	64 (19,3%)	106 (31,9%)	132 (39,8%)	30 (9,0%)

**Abbrevation:** CR: complete response; PR: partial response; SD: stable disease; PD: progression disease

**Table 6:** Response assessment according to mRECIST criteria.

References	N° Pts	CR	PR	SD	PD
**Gillmore R, et al**	83	17 (20%)	31 (37%)	13 (16%)	22 (27%)
**Li H, et al**	64	10 (15,6%)	28 (43,8%)	17 (26,6%)	9 (14,1%)
**Prajapati HJ, et al**	120	24 (20%)	39 (32,5%)	24 (20%)	33 (27,5%)
**Kim BK, et al**	292	117 (40,1%)	93 (31,8%)	73 (25%)	9 (3,1%)
**Jung ES, et al**	114	34 (34,7%)	28 (28,6)	31 (31,6%)	5 (5,1%)
**Kim CJ, et al**	368	162 (44,0%)	88 (23,9%)	51 (13.9%)	67 (18,2%)
**Choi J et al**	332	64 (19,3%)	112 (33,7%)	126 (38,0%)	30 (9,0%)

**Abbrevation:** CR: complete response; PR: partial response; SD: stable disease; PD: progression disease

The number of responders according to mRECIST and EASL criteria was 847 / 1357 (62.4%) and 832 / 1357 (61.3%), respectively.

Kappa statistics (available or calculated by data described in the paper in all the 7 studies) showed very high concordance between responses assessed by using EASL and mRECIST criteria (Table 7). In the pooled population of the 7 studies (Table 8), out of 1357 patients, 1286 agreements were observed between the two methods (94.77% of the observations, kappa statistics 0.928, 95% confidence interval 0.912–0.944).

**Table 7:** Inter-methods concordance between EASL and mRECIST criteria.

References	N° Pts	Treatment	k value
**Gillmore R, et al**	83	TAE/TACE	0,983*
**Li H, et al**	64	Cryoablation	0,91
**Prajapati HJ, et al**	120	DEB TACE	0,82
**Kim BK, et al**	292	TACE	0,863
**Jung ES, et al**	114	TACE	0,883
**Kim CJ, et al**	368	TACE	0,969*
**Choi J, et al**	332	TACE	0,957

*calculated using the data reported in the paper

TAE: trans-arterial embolization; TACE: trans-arterial chemo-embolization; DEB TACE: drug-eluting beads trans-arterial chemoembolization.

**Table 8:** Agreement between mRECIST and EASL response in the 7 studies pooled.

		mRECIST response*				
		CR	PR	SD	PD	
**EASL response** *	CR	424	0	0	0	424
	PR	4	379	25	0	408
	SD	0	40	308	0	348
	PD	0	0	2	175	177
		428	419	335	175	1357

*overall response, *with the exception of the study by Kim et al*., *where only target response was available*. *CR*: *complete response; PR*: *partial response; SD*: *stable disease; PD*: *progressive disease*.

### Survival

With the limitation of the small number of studies included, funnel plots for both EASL and mRECIST estimates did not show asymmetry (S1 Fig and S2 Fig), so there was no clear evidence of publication bias.

**Fig 2:**
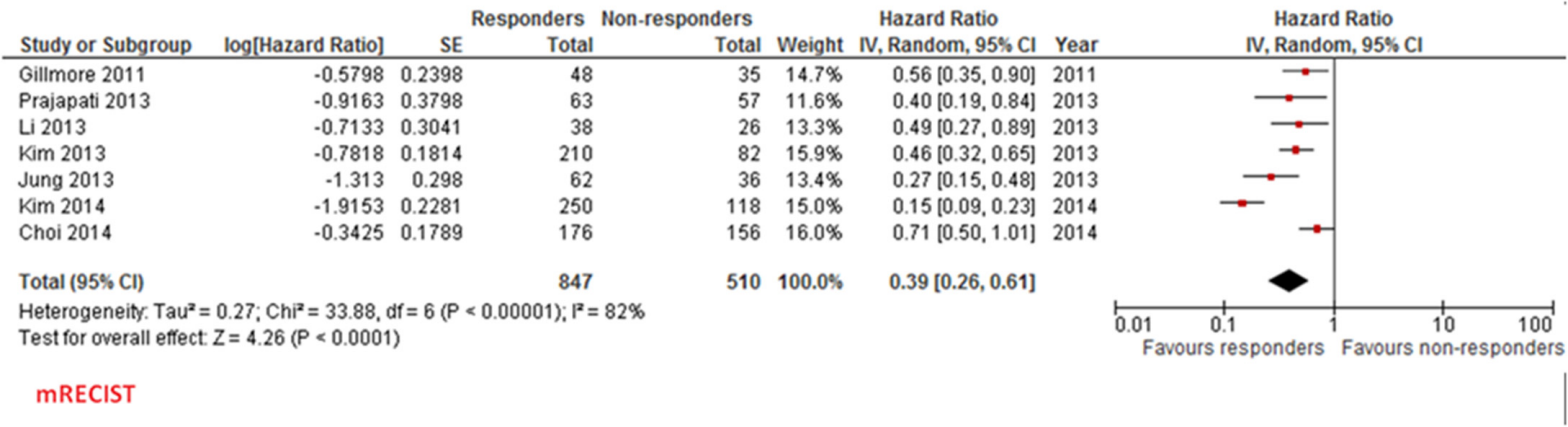
Forest plot for HR for overall survival (responders vs non responders) according to mRECIST criteria.

Hazard Ratio for overall survival (responders versus non responders) according to mRECIST criteria (Fig 2) was 0.39 (95% confidence interval 0.26–0.61, p<0.0001), with a statistically significant heterogeneity among the studies (I^2^ 82%, p<0.00001).

**Fig 3:**
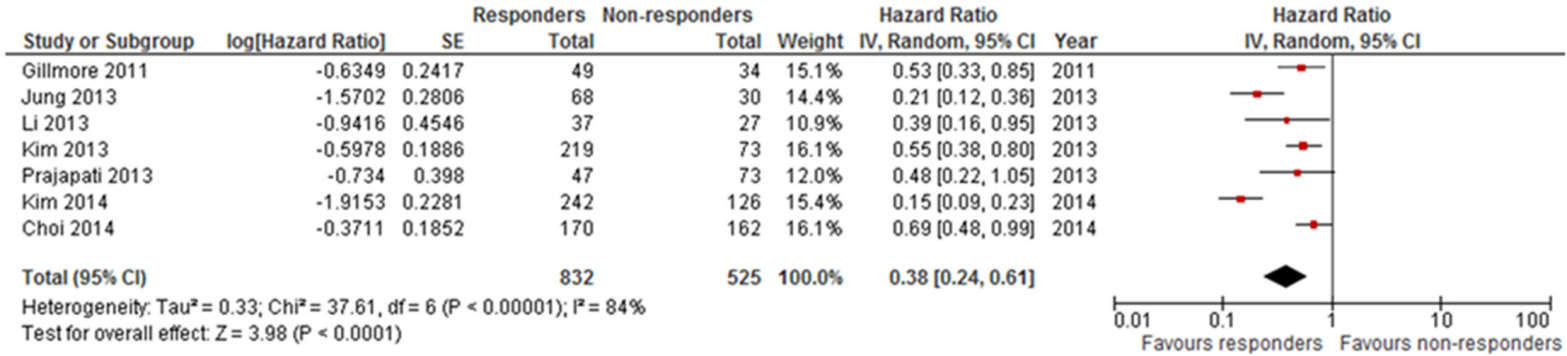
Forest plot for HR for overall survival (responders vs non responders) according to EASL criteria.

Similarly, Hazard Ratio for overall survival (responders versus non responders) according to EASL criteria (Fig 3) was 0.38 (95% confidence interval 0.24–0.61, p<0.0001), with a statistically significant heterogeneity among the studies (I^2^ 84%, p<0.00001).

## Discussion

Tumor response assessment is a cornerstone in cancer patient management, both in everyday clinical practice and as auxillary surrogate end point of survival for the evaluation of treatment efficacy in clinical studies.

Since new biological agents and loco-regional procedures exert their antitumoral activity by inducing tumour necrosis, with rare changes in volume shrinkage, traditional WHO and RECIST criteria do not always represent an appropriate tool for response evaluation, as they are based on dimensional criteria with no indication of lesion density changes. This concept has been very clearly described in several studies where both WHO and RECIST showed poor correlation with survival [8]. This observation, that can be assumed for most of solid cancer, becomes extremely relevant in HCC management, for which antiangiogenic drugs (i.e. sorafenib) and selective ablative procedures represent the standard of care for inoperable disease. To overcome these limitations, the EASL and mRECIST criteria have been suggested to be a better way of assessing tumor response in HCC patients. Several studies, recently reviewed [28], demonstrated their superiority over conventional criteria. The evaluation of tumour viability, represents the most relevant change in EASL/mRECIST criteria compared to traditional WHO/RECIST.

To the best of our knowledge, our meta-analysis is the first comprehensive paper aiming to address the superiority in assessing response of one criterion over the other. Since WHO and RECIST criteria are well recognized as inadequate, we only considered EASL and mRECIST methods and compare their predictivity of survival. Our results show how both EASL and mRECIST response evaluation methods can be of help in predicting long-term survival in HCC patients treated with TACE, with no proven advantage of one method over the other. Our meta-analysis also showed a statistically significant heterogeneity for both mRECIST and EASL criteria. This statistical heterogeneity could be related to relevant differences between the included studies, not only in terms of patients characteristics, but also in the technique used for the treatment procedure, and in the timing of instrumental assessment that, as shown in Table 4, was not the same among the series. However, each single study showed a better prognosis for responders compared to non-responders, with only quantitative heterogeneity in the hazard ratio. Heterogeneity of collection data together with retrospective nature of included studies, represent a large limitation for our work.

Give our results, we believe that EASL and mRECIST criteria deserve further evaluation as response assessment methods in HCC patients undergoing TACE, and that larger prospective trials should be encouraged in the future.

## Conclusion

In this literature-based meta-analysis, mRECIST and EASL criteria showed a very good concordance in HCC patients undergoing loco-regional treatments. Objective response according to both criteria confirm a strong prognostic value in terms of overall survival. This prognostic value appears to be very similar between the two methods.

## Supporting Information

S1 PRISMA Checklist Checklist of preferred reporting items for systematic reviews and meta-analyses (PRISMA) statement. (PDF)

S1 Fig Funnel plot of mRECIST criteria. (PDF)

S2 Fig Funnel plot of EASL criteria. (PDF)
