# Supplementary figures and images for: Prognostic Relevance of Objective Response According to EASL Criteria and mRECIST Criteria in Hepatocellular Carcinoma Patients Treated with Loco-Regional Therapies: A Literature-Based Meta-Analysis
Source: PLoS One. 2015 Jul 31;10(7):e0133488. doi: 10.1371/journal.pone.0133488 (PMC4521926; doi:10.1371/journal.pone.0133488)

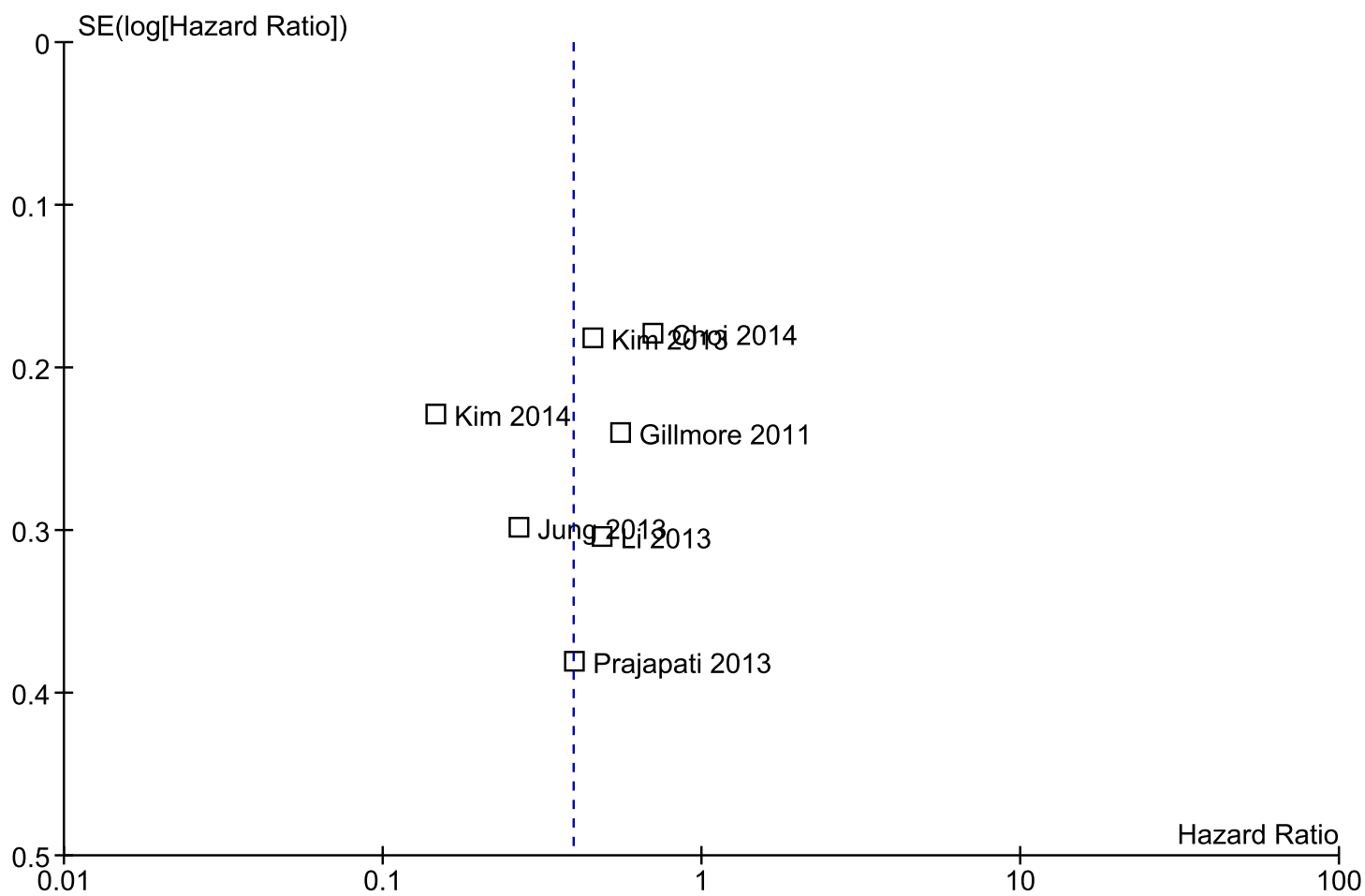

Supplement: S1 Fig — (PDF) [file pone.0133488.s002.pdf]

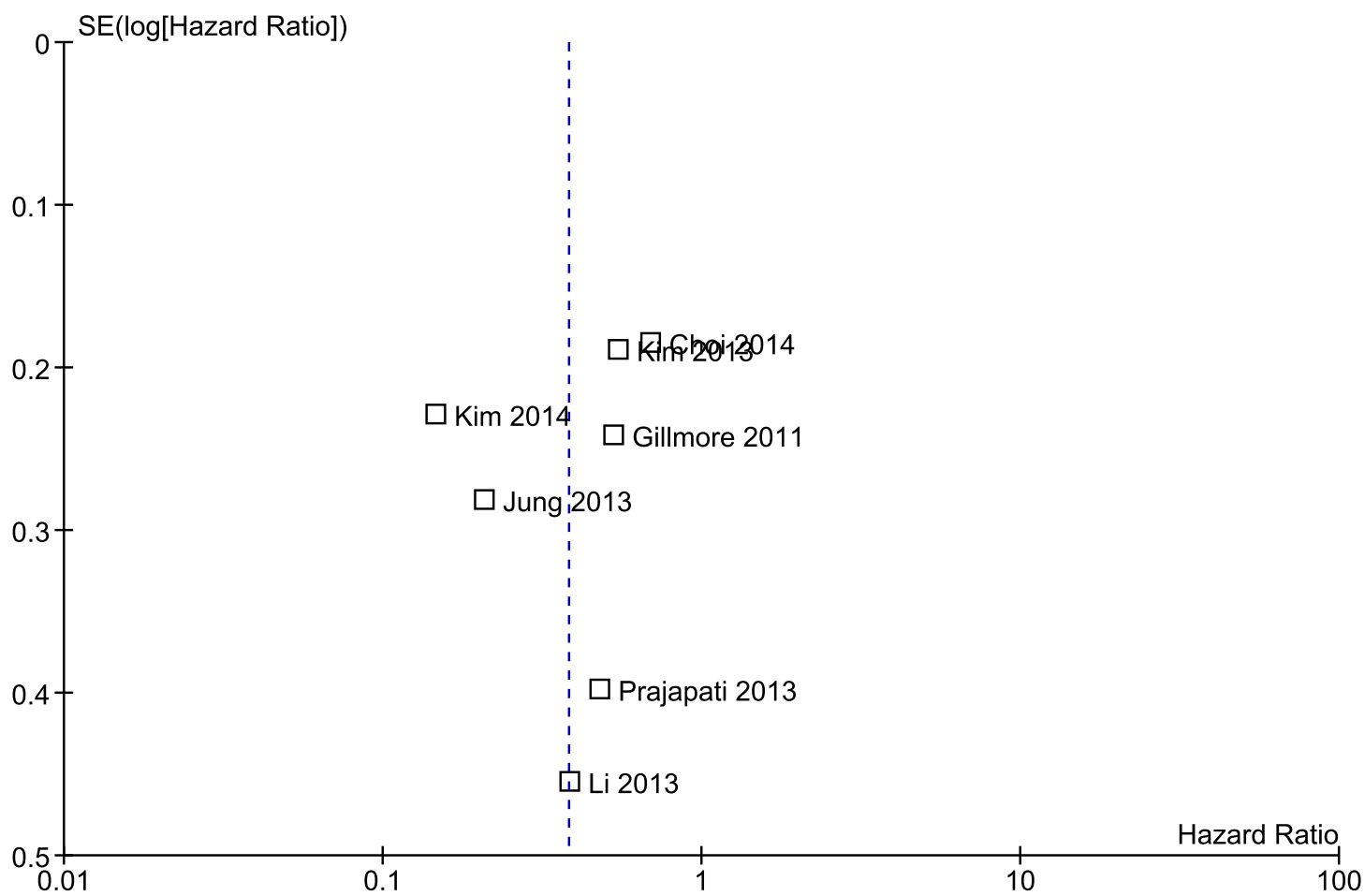

Supplement: S2 Fig — (PDF) [file pone.0133488.s003.pdf]
